# A constant domain mutation in a patient-derived antibody light chain reveals principles of AL amyloidosis

**DOI:** 10.1038/s42003-023-04574-y

**Published:** 2023-02-23

**Authors:** Georg J. Rottenaicher, Ramona M. Absmeier, Laura Meier, Martin Zacharias, Johannes Buchner

**Affiliations:** 1 Center for Functional Protein Assemblies, Technical University Munich, Ernst-Otto-Fischer-Str. 8, 85748 Garching, Germany; 2 Department of Biosciences, TUM School of Natural Sciences, Technical University Munich, Boltzmannstr. 10, 85748 Garching, Germany

**Keywords:** Protein aggregation, Molecular conformation

## Abstract

Light chain (AL) amyloidosis is a debilitating disease in which mutant antibody light chains (LC), secreted by aberrant plasma cell clones, misfold and form insoluble fibrils, which can be deposited in various organs. In the majority of cases, the fibrillar deposits consist of LC variable domains (V_L_) containing destabilizing mutations compared to their germline counterparts. This is also true for the patient LC FOR005. However, this pathogenic LC sequence contains an additional mutation in the constant domain (C_L_). The mechanistic impact of C_L_ mutations is not yet understood in the context of AL amyloidosis. Our analysis reveals that the FOR005 C_L_ mutation influences the amyloid pathway in specific ways: (1) folding and stability of the patient C_L_ domain are strongly impaired; (2) the mutation disrupts the LC dimer interface and weakens dimerization; (3) the C_L_ mutation promotes proteolytic cleavage of the LC monomers resulting in an isolated, amyloidogenic V_L_ domain while dimeric LCs are not cleaved. The enhanced proteolysis rates and the inability of full-length LCs to form amyloid fibrils even in the presence of a destabilized C_L_ domain support a model for AL amyloidosis in which the C_L_ domain plays a protective role and in which proteolytic cleavage precedes amyloid formation.

## Abstract (web summary)

Biophysical approaches reveal how a mutation in the constant light chain domain of a patient-derived antibody destabilizes the constant domain, prevents the dimerization of the light chain, and makes it more prone to proteolytic cleavage, unleashing the amyloidogenic potential of the isolated variable domain.

## Introduction

Light chain (AL) amyloidosis, the most common form of systemic amyloidosis, is a fatal disease in which antibody light chains (LC) misfold and aggregate as highly ordered amyloid fibrils^1^. These insoluble fibrils deposit in various organs, most commonly the heart and kidneys, and cause severe tissue damage^2^. The mutant immunoglobulin LCs that serve as fibril precursors are secreted in large amounts by malignant, monoclonal plasma cells. Therefore, AL amyloidosis is often a comorbidity of plasma cell dyscrasias, such as multiple myeloma^3^. LCs consist of a variable domain (V_L_) and a constant domain (C_L_) which are connected by a short linker^4^. The V_L_ domain is involved in antigen binding during immune responses and is, therefore, subjected to genetic recombination events (VJ-recombination) and somatic hypermutation (SHM) in the process of plasma cell development^5,6^. Thereby, mutations are introduced that lead to a large LC sequence variability, especially in the CDR loops responsible for antigen binding^7^. In AL amyloidosis, the additional mutations that divert the protein to the fibrillary pathway are largely unique for each patient. Of these patient variants, only a limited number has been analyzed on the protein level. These analyses have revealed the presence of “active”, fibril-promoting mutations, and “silent” ones which do not induce the fibril pathway^8^. One of the well-characterized AL variants is FOR005, which was derived from a patient with cardiac involvement. As in many cases of AL amyloidosis, FOR005 fibrils contained only the V_L_ domain which belongs to the λ3l subtype^9^. Comparison with the closest germline sequence revealed five mutations in the patient V_L_ domain. Two out of the five V_L_ point mutations were shown to be causative for destabilization and amyloid formation of the isolated patient V_L_. Interestingly, these two decisive mutations are located in the hypervariable CDR2 and CDR3 loops of the V_L_ domain and strongly influence the conformational dynamics of the surrounding framework regions^10^. However, FOR005 also contains a nonconservative valine to glycine mutation in its C_L_ domain. Generally, the role of the C_L_ in AL amyloidosis still remains elusive, although amyloid deposits containing C_L_ domains have been reported^11–13^. Since the C_L_ domain is normally not affected by the aforementioned mechanisms of VJ-recombination and hypermutation, amino acid substitutions in this region are quite rare and not well characterized^14,15^. Thus, it was not clear whether the FOR005 C_L_ mutation is an “active” or “silent” mutation. If it were active, this would raise the question of how it influences fibril formation despite not being present in the fibril itself. Since these are fundamental open questions in the context of AL amyloidosis, we analyzed the effect of this substitution on the biochemical and biophysical features of the full-length LC and the C_L_, respectively. We found that the V136G mutation affects the C_L_ domain and the full-length LC by impairing folding, thermodynamic stability, and dimerization. In consequence, the sensitivity for proteolytic cleavage is enhanced resulting in a free, amyloidogenic V_L_ domain.

## Results

### The sequence of the FOR005 LC contains an unusual constant domain mutation

**Fig. 1:**
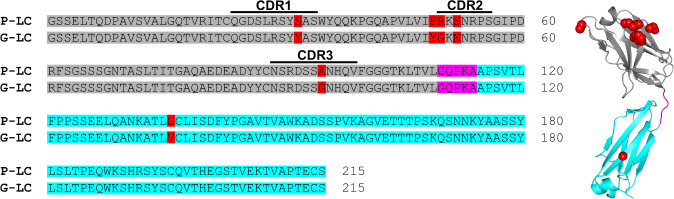
Sequence alignment and homology model of FOR005. The V_L_ domain is colored in gray, the linker region in magenta, and the C_L_ domain in cyan. Mutations are indicated in red. The homology model was created with SWISS-MODEL using template structure 5BV7. The C-terminal cysteine at position 214 has been mutated to serine in all constructs to result in monomeric proteins.

The FOR005 LC sequence (subtype λ3l; gene segments: IGLV3-19*01, IGLJ2*01, *IGLC2*) was obtained from a patient suffering from a λ-restricted monoclonal gammopathy and heart failure due to AL amyloidosis. The patient-derived amyloid deposits consist of the V_L_ domain only^9^. We used the databases IMGT, abYsis, and IgBLAST to identify the most closely related germline LC amino acid sequence (G-LC) and found that the patient LC (P-LC) contains six mutations in total (Fig. 1)^16–18^. Five of the six mutations are found in the V_L_ domain and four of them are in the hypervariable complementarity determining regions (CDRs). These five V_L_ domain mutations were analyzed in a previous study, showing that G49R and G94A are the key drivers of destabilization and fibrillary aggregation of the FOR005 V_L_ domain^10^. However, FOR005 also carries a point mutation in the C_L_ domain which is quite rare. Conservation analysis of the G-LC using ConSurf showed the valine at position 136 (valine 133 in the Kabat numbering scheme)^19^ to be highly conserved and buried which implies an integral structural role (Supplementary Fig. 1)^20^. Further, we applied homology modeling using SWISS-MODEL (template structure: 5BV7) and observed that position 136 is located in the middle of the C_L_ β-strand b which is part of the highly conserved β-sandwich topology of Ig domains^21^. Conclusively, a nonconservative substitution that alters side chain properties at this position is likely to have a strong effect on the biophysical and biochemical characteristics of the C_L_ domain and, therefore, on the entire LC.

### The C_L_ mutation V136G alters structural properties and thermodynamic stability

**Fig. 2:**
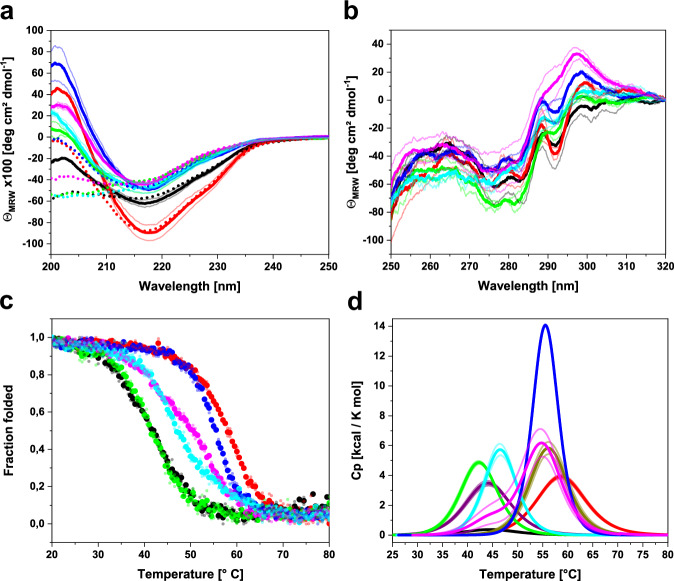
Structural properties and thermodynamic stability of FOR005 variants. **a** FUV-CD spectra of the native (solid lines) and refolded (dotted lines) proteins indicating secondary structure. The mean of two measurements is shown as thick lines, and the spectra of individual replicates are shown as thin lines with weaker coloring. Color code: P-CL in black, G-CL in red, P-LC in green, G-LC in blue, P-LC G136V in cyan, G-LC V136G in magenta. **b** NUV-CD spectra of the native proteins representing tertiary structure fingerprints. Coloring as in (**a**). **c** Normalized thermal unfolding transitions followed by CD at 205 nm. The transition midpoint T_m_ indicates the melting temperature at which 50% of the protein is unfolded. Mean values are shown as thick dots and individual data points as smaller dots with light coloring. **d** Thermograms obtained by DSC. For comparison, the P-VL (purple) and G-VL (olive) were also included. Mean curves are shown as thick lines, individual measurements are shown as thin lines with weak coloring. All data are derived from two independent measurements (*n* = 2), except for the refolded FUV-CD spectra after thermal unfolding (dotted lines in panel (**a**)) which were only measured once. Source data are given in the Supplementary Data file.

To decipher the role of the constant domain mutation we investigated the patient C_L_ (P-CL), the germline C_L_ (G-CL), and the respective patient and germline LCs (P-LC, G-LC). Additionally, we created the chimeric mutants P-LC G136V (patient V_L_ + germline C_L_) and G-LC V136G (germline V_L_ + patient C_L_) to obtain more detailed insight into the interplay between the two LC domains. In all of these constructs, the C-terminal cysteine residue at position 214—normally involved in the covalent linkage of LC and heavy chain (HC) or LC homodimers via disulfide bond^22^—was mutated to serine resulting in monomeric LCs. All proteins were recombinantly produced as inclusion bodies in *E. coli*, refolded, and purified. To assess the secondary structure, far-UV circular dichroism spectroscopy (FUV CD) was performed (Fig. 2a). All constructs exhibit spectra indicative of a β-sheet structure, typical for immunoglobulin domains^23^. However, the secondary structure content varies between the different proteins as can be seen by the differences in molar ellipticity around 200 nm. Especially the P-CL (black curve) exhibits a decreased signal, which points toward a larger portion of disordered segments or partial unfolding. The dashed lines represent spectra of refolded proteins after thermal unfolding. The G-CL (red curve) and G-LC (blue curve) regain their native structure to a large extent after unfolding but not completely. However, it should be noted that thermal unfolding is often accompanied by aggregation which could explain the signal discrepancy between the spectra of the native and refolded germline variants^24^. The remaining proteins, which contain patient-specific mutations in either of the two domains, stay largely unfolded after thermal denaturation and refolding, indicating irreversible unfolding (Fig. 2a). Near UV (NUV) CD spectra represent a protein-specific tertiary structure fingerprint. Since all NUV-CD spectra look similar, it can be concluded that the overall structural topology does not differ strongly between the different LCs and C_L_s (Fig. 2b). Therefore, the main differences observed by CD seemingly lie in local secondary structure elements. To investigate protein stability, thermal denaturation curves between 20 and 80 °C followed by CD at 205 nm were recorded (Fig. 2c, Table 1). The transition midpoint (T_m_) represents the melting temperature at which 50% of the protein is unfolded. The T_m_ values for the patient and germline V_L_ domains (P-VL and G-VL) were taken from our previous study for comparison^10^. As expected, the patient C_L_, V_L_, and LC exhibit considerably lower thermal stability compared to their respective germline counterparts with melting point differences (ΔT_m_) ranging between ~13 °C and 16 °C (Table 1). As for the two chimeric LCs, the P-LC G136V variant is stabilized by ~6 °C compared to the P-LC, whereas G-LC V136G is destabilized by roughly 6 °C in comparison to G-LC (Table 1). Thus, the chimeric mutants exhibit intermediate T_m_ values lying in between the stabilities of P-LC and G-LC, which correlates well with the secondary structure content of the LCs (Fig. 2, Table 1). Of note, the derived melting temperatures represent apparent values since the proteins do not unfold reversibly.

**Table 1:** Thermodynamic stability parameters determined by thermal unfolding using CD spectroscopy at 205 nm and DSC.

Protein	T_m_ by CD	T_m_ by DSC	ΔH_cal_	ΔH_VH_
	°C	°C	kcal/mol	kcal/mol
P-CL	41.04	44.55	5.19	58.95
G-CL	57.78	58.80	52.80	66.60
P-VL	43.50^a^	44.15	42.50	65.65
G-VL	56.30^a^	56.22	58.95	85.75
P-LC	41.38	42.34	48.25	79.95
G-LC	55.28	55.58	97.00	124.5
P-LC G136V	47.46	46.57	49.90	93.35
G-LC V136G Peak 1	49.35	44.44	10.41	84.55
G-LC V136G Peak 2		54.92	62.40	84.45

All experiments were carried out in duplicates (*n* = 2) and mean values are reported. CD data were analyzed applying a Boltzmann fit, for DSC data a non-two-state fitting model was used.

^a^T_m_ values by CD of FOR005 P-VL and G-VL were taken from ref. ^10^

To characterize the thermodynamic stability of the variants in more detail, we performed differential scanning calorimetry (DSC). This method allows the determination of the apparent T_m_ and of the unfolding enthalpy either calorimetrically (ΔH_cal_) or via the van't Hoff relation (ΔH_VH_) by applying a non-two-state fit model^25^. The melting temperatures obtained for the constructs by DSC match the values derived from the thermal unfolding experiments monitored by CD spectroscopy (Fig. 2d, Table 1). Generally, all proteins tested unfold in a single transition except for G-LC V136G, which shows separate unfolding peaks for the two constitutive domains (Fig. 2d). For P-LC and G-LC it seems plausible that the T_m_ values of the individual domains are too close to each other to be resolved by DSC. In the case of P-LC G136V, however, based on the results for the individual domains, the two domains should have quite different melting points, yet this LC still unfolds in a single transition which indicates a cooperative interplay between the two domains. We assume that this cooperative unfolding is not observable in the G-LC V136G due to the loss of V_L_–C_L_ interactions which are disrupted in the mutant. Furthermore, in DSC experiments ΔH_cal_ and ΔH_VH_ should be identical if the protein exhibits an ideal two-state unfolding transition^26^. Discrepancies between the two enthalpy values point toward the existence of intermediate states along the unfolding pathway that diverts the unfolding reaction from a simple two-state mechanism^27^. We determined the ΔH_cal_/ΔH_VH_ ratios for all samples and observed that the values of all germline proteins (G-CL, G-VL, G-LC) tend to be closer to 1 than the ratios of the proteins which carry patient mutations (P-CL, P-VL, P-LC, P-LC G136V, G-LC V136G) (Supplementary Fig. 2). This implies that the mutations in both the patient V_L_ and patient C_L_ favor the population of partially unfolded states along the folding trajectory. Such folding intermediates are known to play a key role in the amyloid pathway of LCs and V_L_s^28^.

### The mutation V136G induces partial unfolding of the C_L_ domain

**Fig. 3:**
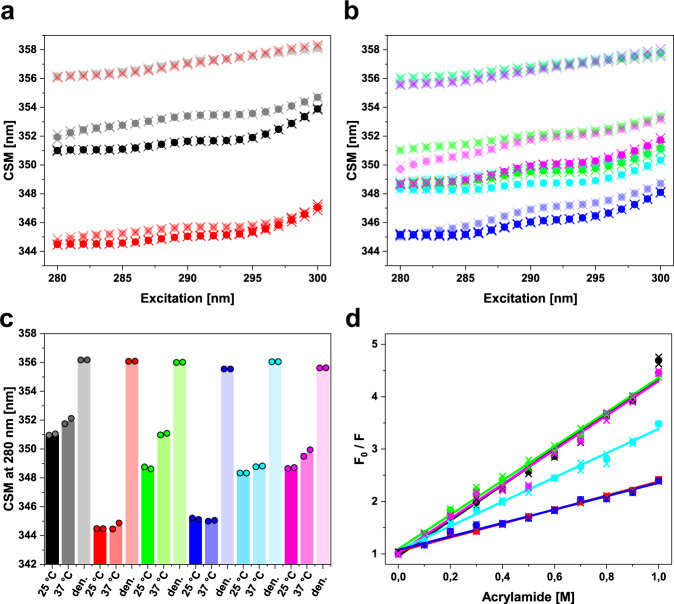
REES fluorescence and acrylamide quenching give insights into folding and conformational flexibility. **a** REES data of P-CL (black) and G-CL (red) at 25 °C (solid), 37 °C (light coloring), and in the presence of 6 M urea (very light coloring; data points at the top). The CSM value at 280 nm indicates solvent exposure of the tryptophan fluorophores. Mean values are shown as dots, and individual data points are shown as crosses. **b** REES data of P-LC (green), G-LC (blue), P-LC G136V (cyan), and G-LC V136G (magenta). Data at 25 °C are shown as solid dots, data at 37 °C as light dots, and data with 6 M urea in very light coloring (points at the top). Individual data are shown as crosses. **c** Bar graph of the CSM values at 280 nm for all constructs under the three conditions tested. The data in the presence of 6 M urea is termed den. for “denatured”. Coloring according to (**a**) and (**b**). The bars represent the mean value, individual data points are shown as circled dots. **d** Acrylamide quenching plots showing F_0_/F as a function of acrylamide concentration, where F_0_ is the native fluorescence. Coloring is the same as in (**a**), (**b**), and (**c**). Mean values are shown as dots, and individual data points are shown as crosses. The slope of the linear fit represents the Stern–Volmer constant k_sv_ (Table 2). For G-LC V136G (magenta), the data point at 0.5 M was excluded from the fit. The fluorescence data are derived from two independent experiments (*n* = 2). Source data are given in the Supplementary Data file.

To gain further insight into the folding and conformational properties of the LCs and especially the mutated C_L_ domain, we used intrinsic tryptophan fluorescence, specifically red-edge excitation shift (REES) and acrylamide quenching experiments (Fig. 3, Table 2). In tryptophan fluorescence spectra (excitation wavelength = 295 nm), we observed a red shift in the emission maxima and increased fluorescence intensities for constructs carrying patient mutations (Supplementary Fig. 3). These spectral features hint at partial unfolding, especially regarding the striking difference between the isolated G-CL and P-CL domain spectra (Supplementary Fig. 3). REES experiments of P-CL and G-CL at 25 and 37 °C showed the increased center of spectral mass values (CSM) for P-CL which indicates higher solvent exposure of the tryptophan fluorophores and, therefore, also a higher degree of partial unfolding (Fig. 3a). Further, the effect of elevated temperature (25 vs. 37 °C) is more pronounced in P-CL than in G-CL (Fig. 3a). Regarding the LCs, the G-LC shows the lowest CSM value, as expected (Fig. 3b). P-LC and the two chimeric mutants all exhibit very similar REES profiles at 25 °C although the difference between starting and end values (ΔCSM = CSM_300_ − CSM_280_) differs between the curves indicating differences in conformational dynamics^29^. In addition, the shift toward higher CSM values is more pronounced at 37 °C for P-LC and G-LC V136G, which demonstrates the beneficial effect of valine 136 on the stability and dynamics of the C_L_ domain (Fig. 3b, c; Table 2).

**Table 2:** CSM values at *λ*_ex_ = 280 nm determined by REES fluorescence spectroscopy and Stern–Volmer constants (k_sv_) derived from acrylamide quenching experiments.

Protein	CSM_280_ at 25 °C	CSM_280_ at 37 °C	k_sv_ at 25 °C
	nm	nm	L/mol
P-CL	351.00	351.93	3.39
G-CL	344.47	344.65	1.33
P-LC	348.68	351.02	3.27
G-LC	345.16	345.02	1.28
P-LC G136V	348.32	348.79	2.32
G-LC V136G	348.67	349.71	3.28

All experiments were carried out as duplicates (*n* = 2) and the mean values of the two experiments are reported.

Acrylamide quenching can be used to investigate the accessibility of fluorophores and thus can provide insights into domain architecture and dynamics^30^. The Stern–Volmer constant (k_sv_) is the slope of the linear fitting curve and represents the degree to which tryptophan fluorescence is quenched (Fig. 3d, Table 2). G-CL and G-LC expectedly show the lowest degree of quenching which correlates to their conformational stability. The proteins P-CL, P-LC, and G-LC V136G (i.e., all constructs with a mutated, destabilized C_L_ domain) exhibit very similar quenching curves and k_sv_ values (Fig. 3d, Table 2). The value for P-LC G136V lies in between. Thus, the increased quenching compared to the two germline constructs can be attributed to the effects of the patient-specific mutations in the V_L_ domain. In conclusion, the fluorescence data imply partial unfolding of the P-CL and further demonstrate the negative effects of the V136G mutation on the FOR005 patient C_L_.

**Fig. 4:**
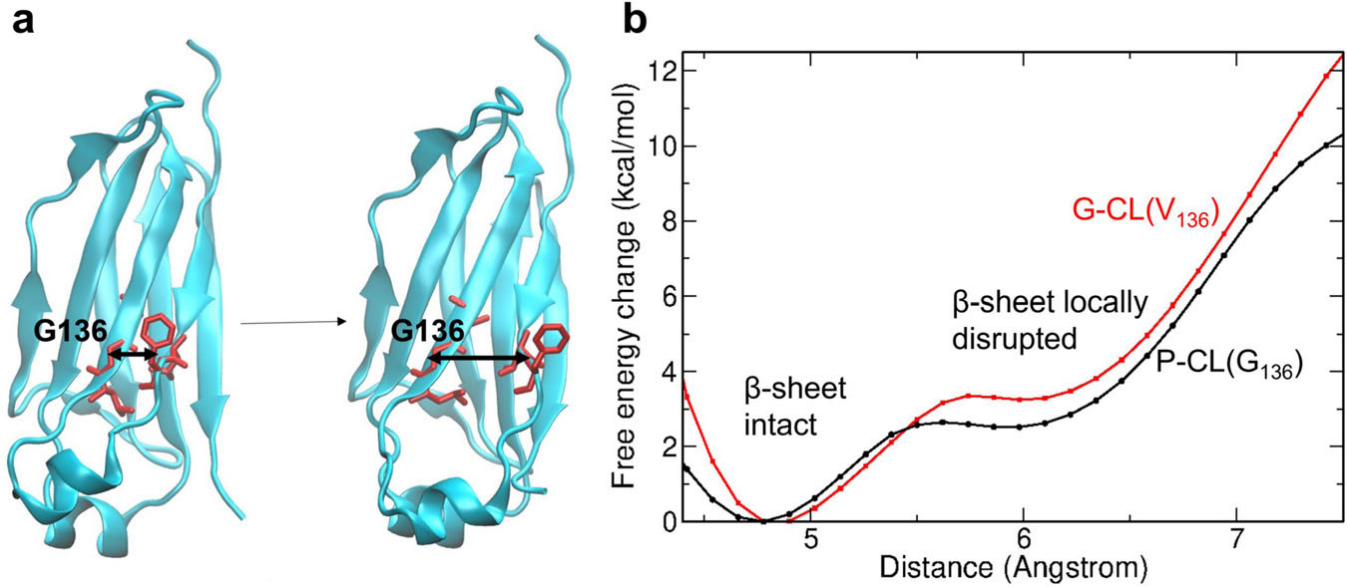
Free energy simulations on P-CL and G-CL. **a** Umbrella sampling free energy simulations on partial dissociation of the N-terminal β-strand near the location of residue 136 were performed on the P-CL and G-CL (cartoon illustration) variants. The center of mass (com) distance between backbone atoms of residues 135–137 and residues 120–121 (red sticks) served as the reaction coordinate (double arrow). **b** Calculated free energy changes (potential of mean force) along the reaction coordinate for P-CL (black line) and G-CL (red line). Single simulations were performed (*n* = 1). Source data are given in the Supplementary Data file.

In addition, we performed molecular dynamics (MD) simulations on the P-CL and G-CL variants. Starting from the folded domains, no unfolding or any other major conformational change was observed on the timescale of the MD simulations (500 ns). Both P-CL and G-CL resulted in similar root-mean-square deviation (RMSD) from the start structure and root-mean-square fluctuations (RMSF) (Supplementary Fig. 4). Residue 136 is located next to a cysteine that is involved in the single disulfide bond in the C_L_ domain and forms backbone hydrogen bonds to the N-terminal β-strand. In order to investigate if the greater conformational flexibility of a G136 may affect the interaction with the N-terminal β-strand, which in turn may affect the stability, we performed free energy simulations to partially dissociate the N-terminal β-strand and disrupt the backbone hydrogen bonds with the residues 135–137 (Fig. 4). Indeed, the calculated free energy penalty to locally disrupt the β-sheet segment is lower for the P-CL variant by ~1 kcal/mol. This indicates that the V136G mutation in the C_L_ domain destabilizes the β-sheet segment near the N-terminus, which contributes to the lower stability of the P-CL vs. G-CL.

### The full-length LC and C_L_ domain of FOR005 are resistant to amyloid formation in vitro

As mentioned above, in the majority of cases the fibrillar deposits contain mainly the V_L_ domain. There are only a few cases where full-length LCs or C_L_ domains undergo amyloid formation^1,8^. The FOR005 fibrils isolated from patient tissue contain only the V_L_ domain and the amyloid propensity of the patient V_L_ domain was demonstrated in vitro^9,10^. In this study, we tested whether the P-LC or the G-LC form amyloid fibrils in vitro at a physiological pH of 7.4. Further, we investigated whether the germline or patient C_L_ domains and LCs form fibrils not only under native but also under destabilizing conditions. We performed Thioflavin T (ThT) binding assays under continuous shaking at pH 7.4 or 6.4 or in the presence of 0.5 mM SDS which can accelerate fibril formation^31^. However, we did not observe fibril formation for any of the constructs under the conditions tested despite their low thermodynamic stabilities (Supplementary Fig. 5). Transmission electron microscopy (TEM) of the samples revealed that the proteins form amorphous aggregates instead of fibrils (Supplementary Fig. 5). Thus, the presence of the C_L_ domain and its interactions with the V_L_ domain alter the folding pathway and seemingly prevent the LCs from accessing the fibrillary state. Although the patient C_L_ domain exhibits significantly lower stability, it still exerts a protective function, which favors amorphous aggregation over fibril assembly.

### Proteolytic cleavage of LCs is affected by an unstable C_L_ domain

**Fig. 5:**
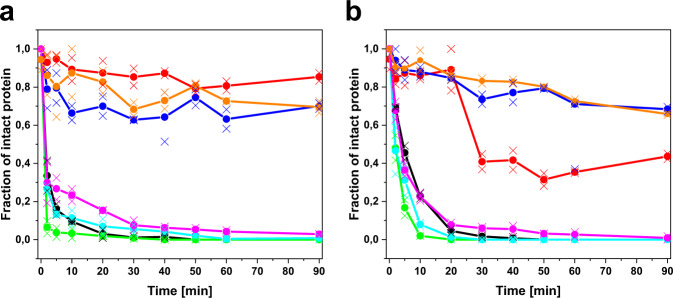
Limited proteolysis reveals the influence of the C_L_ mutation on LC cleavage. **a** Limited proteolysis at room temperature with trypsin (substrate/protease = 20/1). Coloring: P-CL in black, G-CL in red, P-LC in green, G-LC in blue, P-LC G136V in cyan, G-LC V136G in magenta, covalent P-LC homodimer (Bence-Jones dimer) in orange. The mean values are shown as connected dots and the individual data points as crosses. **b** Limited proteolysis with proteinase K at room temperature (substrate/protease = 250/1). Coloring and symbols as in (**a**). The data are derived from two independent experiments (*n* = 2); the single data point of G-LC at 60 min in panel (**b**) was neglected. Source data are given in the Supplementary Data file.

The finding that the FOR005 P-LC does not form fibrils in vitro whereas the patient V_L_ domain does, implies that the LC needs to be cleaved in order to set the destabilized V_L_ domain free. In general, the proposed proteolytic cleavage as a prerequisite for fibril formation is still subject to debate in AL amyloidosis^8^. For FOR005, we applied limited proteolysis to address this question. The LC proteins were incubated with the model proteases trypsin or proteinase K and we followed the degradation kinetics by SDS-PAGE (Fig. 5, Supplementary Fig. 6). We observed that the G-LC is cleaved far more slowly compared to constructs carrying patient mutations in either of the two domains (Fig. 5): the P-LC was cleaved completely within a few minutes, whereas the two chimeric mutants P-LC G136V and G-LC V136G showed slightly increased proteolytic stability although they are also cleaved completely within 90 min. Since we suspected the mutated LCs to be cleaved in the linker region between V_L_ and C_L_ domain—thus setting free the amyloidogenic patient V_L_—we also combined limited proteolysis of P-LC and G-LC V136G with mass spectrometry (LC-MS) to characterize the resulting proteolytic fragments (Supplementary Fig. 7, Supplementary Table 1). Analysis of the LC-MS data supports the notion that proteolytic cleavage of the LC occurs in the linker region that connects V_L_ and C_L_ and that the patient-specific V136G mutation favors that cleavage (Supplementary Table 1).

Furthermore, we also tested proteolysis of the P-CL and G-CL since limited proteolysis can also give information about conformational dynamics. We observed very rapid and complete degradation of the P-CL compared to the G-CL, which is indicative of high structural flexibility and further supports our hypothesis that the isolated P-CL is partially unfolded. Of note, the experiment with G-CL and proteinase K yielded a rather unusual time course with a large, rapid drop in the intact protein fraction down to ~40% rather than a continuous exponential decrease. However, we observed no complete degradation of G-CL by proteinase K, i.e., a large portion of the protein remained uncleaved after 90 min (Supplementary Fig. 6).

The proteolysis data clearly show the mutation-dependent differences in susceptibility for the monomeric LCs. However, in vivo LCs from plasma cells can exist both as monomers and as covalent, disulfide-linked homodimers, so-called Bence-Jones dimers^32^. Therefore, we created a construct in which the C-terminal cysteine residue of the P-LC was re-introduced to allow dimer formation during in vitro refolding. By refolding from inclusion bodies, we obtained a mixture of P-LC dimers and monomers (roughly a 50/50 distribution) which allowed us to determine their susceptibility to degradation in the same experiment. When we performed the limited proteolysis experiments with the P-LC dimer-monomer mixture, we observed that the covalent P-LC dimer is far more resistant to proteolytic cleavage than the monomeric P-LC (Fig. 5, Supplementary Fig. 6). Therefore, covalent dimerization exerts a protective function implying that in vivo not the Bence-Jones dimer, but primarily the monomeric LC of FOR005 is responsible for the proteolytic release of the amyloidogenic V_L_ domain. In this context, characterization of the P-LC dimer-monomer mixture by CD spectroscopy also revealed minor changes in structure, folding, and stability as a result of the covalent dimerization via the disulfide bridge (Supplementary Fig. 8). In conclusion, both the V_L_ and C_L_ mutations favor proteolytic cleavage of the monomeric patient LC that, in the case of FOR005, appears to be a prerequisite for amyloid aggregation. Strikingly, the covalent P-LC dimer is protected from proteolytic cleavage in vitro, which potentially relies on small structural changes due to covalent dimerization.

### The C_L_ mutation impairs homodimerization

**Fig. 6:**
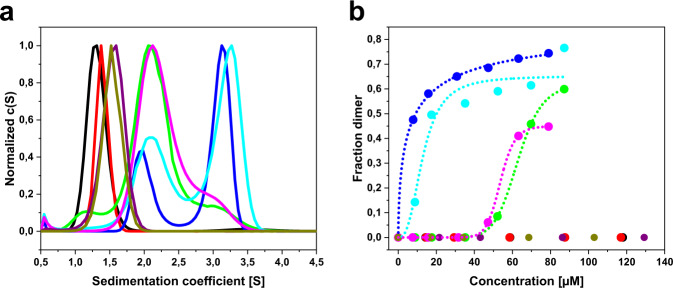
Analytical ultracentrifugation shows that the C_L_ mutation leads to diminished dimerization propensity. **a** Normalized sedimentation profiles of P-CL (black), G-CL (red), P-VL (purple), G-VL (olive), P-LC (green), G-LC (blue), P-LC G136V (cyan), and G-LC V136G (magenta). Protein concentration was adjusted to an absorbance of A_280_ = 1.5. AUC raw data were analyzed with SEDFIT. **b** Fraction of noncovalent dimers dependent on protein concentration. The coloring scheme is the same as in (**a**). Data were fitted using the Hill equation to determine the apparent K_D_ values of the noncovalent LC dimerization. The fits were constrained to include a dimer fraction of 0 at 0 µM protomer concentration. All AUC data are derived from single measurements at each LC concentration (*n* = 1). The corresponding dimer and monomer percentages at each LC concentration are given in Supplementary Table 2.

Apart from stability, partial unfolding, and proteolytic cleavage, the LC quaternary structure also plays a key role in the onset of AL amyloidosis^8^. As mentioned above, free LCs can either be secreted by plasma cells as covalently linked homodimers, so-called Bence-Jones proteins, or as monomeric LCs^32^. We therefore investigated the dimerization propensity of FOR005 constructs by analytical ultracentrifugation (AUC) at varying protein concentrations (Fig. 6, Supplementary Fig. 9, Supplementary Table 2). In all proteins analyzed by AUC, the C-terminal cysteine residue was mutated to serine, so that no intermolecular disulfide formation could occur. Thus, only noncovalent association was monitored reflecting the interactions of the dimer interfaces. We observed that both isolated C_L_ domains and V_L_ domains stay monomeric regardless of the concentration used (Fig. 6). Of the four full-length LCs, P-LC shows the lowest dimerization propensity with an apparent K_D_ of 62.92 µM (Fig. 6, green curves). In contrast, G-LC preferentially forms noncovalent dimers already at low concentrations with a significant shift toward dimer formation with increasing concentrations. The apparent K_D_ of G-LC is 6.09 µM (Fig. 6, dark blue). The chimeric mutant P-LC G136V also exhibits a concentration-dependent monomer–dimer equilibrium with a K_D_ of 12.97 µM, thus the propensity of homodimerization is lower than in G-LC. Regarding the variant G-LC V136G, we only observed dimerization at relatively high protein concentrations, similar to the behavior of P-LC, resulting in an apparent dimerization K_D_ of 53.67 µM. These results suggest that the V136G mutation disrupts the dimer interface and thereby prevents homodimerization. However, our data also imply that both V_L_ and C_L_ domains contribute to the dimer interface since dimerization of the P-LC G136V variant is not fully restored when compared to G-LC and G-LC V136G shows higher dimerization propensity than P-LC. Thus, the patient-specific mutations in the V_L_ domain also diminish the dimerization propensity, although the impact of the C_L_ domain on the quaternary structure is more pronounced.

## Discussion

AL amyloidosis is a challenging disease as each patient seems to have a unique set of mutations. Accordingly, there are still many open questions about the mechanisms that underlie AL onset and progression. The large sequence diversity of precursor LCs also complicates the development of novel diagnostic and therapeutic approaches^8^. Further, the amyloid deposits in patients can contain either entire LCs or different fragments thereof, i.e., the V_L_ or the C_L_^1^. This raises the question of when proteolytic cleavage takes place and which factors determine the occurrence of such endoproteolysis events^8^. In the case of FOR005, the patient’s amyloid fibrils are made up of the V_L_ domain only^9^. Therefore, in the first study, we focused on the influences of individual point mutations on the characteristics of the V_L_ domain. We were able to show that the point mutations in hypervariable CDR loops affect the dynamics of surrounding framework regions, which destabilizes the V_L_ domain and increases its aggregation propensity^10^. FOR005, however, also carries a mutation in the conserved C_L_ domain, which is rare since it is usually not subject to somatic hypermutation during B cell maturation^22^. Therefore, we set out to characterize the influence of this C_L_ mutation on the properties of the LC and its involvement in disease onset.

The ConSurf web tool identified the valine at position 136 of the LC to be highly conserved and to be a buried residue which means it is most likely an integral residue for the protein’s structure and stability^20^. Thus, we expected that mutation of the valine to glycine, as it occurs in the patient’s LC, could have a pronounced effect on the properties of the LC. Indeed, we found the structure and the stability of the LCs containing the mutation and of the isolated P-CL to be strongly affected. The CD spectra show reduced secondary structure content for variants containing the V136G substitution and decreased thermodynamic stability. Further, unfolding reversibility after thermal unfolding is impaired by the presence of the C_L_ mutation. Comparative MD free energy simulations on the P-CL and G-CL domains indicate a larger free energy penalty of disrupting the β-sheet interaction of residues around G136 with the N-terminal β-strand compared to the G-CL with a V136. However, the calculated magnitude of the effect may not be sufficient to explain the significantly lower stability of the P-CL vs. G-CL. It might be possible that the G136 mutation in P-CL allows the formation of an increased number of stable unfolded or misfolded conformations that overall shift the equilibrium toward unfolded states. Such states cannot be sampled on the timescale of the present MD simulations. Despite the structural alterations and the resulting destabilization, the mutant C_L_ still exerts a protective function regarding amyloid fibril formation. In our ThT assays, none of the LC constructs formed fibrils even under destabilizing conditions (lowered pH, addition of SDS) while the patient V_L_ readily forms fibrils in vitro^10^. This phenomenon has been reported for other cases before^33,34^, yet, there are also cases where full-length LCs readily aggregate into fibrils^35–37^. In the case of FOR005, however, all LC variants and both C_L_s formed only amorphous aggregates as visualized by TEM. Therefore, the presence of the C_L_ domain diverts the LC from the amyloid pathway in favor of amorphous aggregation as these two pathways of protein aggregation can be viewed as competing processes^38^.

This protective effect of the C_L_ domain likely relies on interdomain contacts between C_L_ and V_L_ which play a key role regarding the biophysical properties of LCs^39^. Rennella et al. have shown that V_L_–C_L_ interactions can have a strong influence on the unfolding and aggregation pathway of an LC and that even a destabilized and partially unfolded C_L_ can still protect the LC from amyloid formation, or at least slow down the process considerably^40^. A previous study further demonstrated that amyloidogenic mutations in the V_L_ domain can shift V_L_–C_L_ interactions from native to nonnative thereby destabilizing the entire LC and abolishing the C_L_’s protective function^41^. In the present study, we also observed the effects of the V_L_–C_L_ interactions and showed that partial unfolding of the mutated C_L_ domain abrogates interdomain contacts and their cooperative behavior as seen by the DSC experiments. However, even when the domain interactions are lost, the LCs do not enter the fibril pathway which could be explained by the C_L_’s low intrinsic amyloid propensity which outweighs that of the V_L_^40^.

The importance of interdomain contacts also becomes apparent when proteolysis is considered. The general importance of proteolytic cleavage of LCs in AL amyloidosis is still subject to debate but increasing evidence points toward a case- and sequence-dependent phenomenon^8^. The FOR005 P-LC monomer and the two chimeric mutants are readily cleaved by model proteases. The sizes of the resulting fragments correspond to the two constituent domains of the LC, which means that cleavage occurs in the linker region. Therefore, it seems plausible that interdomain contacts also govern the accessibility of the LC linker for endoproteases since proteolysis is observed as soon as one of the two domains carries destabilizing substitutions. Only G-LC, in which neither of the domains is mutated, is resistant to proteolytic cleavage. This means, that no matter how intrinsically stable the V_L_ or C_L_ is, it cannot prevent cleavage of the full-length LC if the other domain is sufficiently destabilized by mutations. Another explanation involves the population of partially folded intermediates since Morgan and coworkers demonstrated that partially folded LCs are far more prone to proteolytic cleavage than natively folded ones and that transient unfolding and incomplete refolding of LCs can enable endoproteolysis^42^. Our DSC data show that P-LC and both chimeric mutants are less likely to exhibit two-state unfolding meaning they more readily populate folding intermediates. If one of the domains carries unfavorable mutations, the population of partially folded intermediates is enhanced, kinetic stability is decreased, and proteolysis can occur more easily. In conclusion, the mutations of the V_L_ and C_L_ strongly favor the proteolytic processing of FOR005 P-LC giving rise to its amyloidogenic V_L_ domain.

Proteolytic cleavage appears to play a major role in the disease pathway of FOR005. However, in the case of FOR005, it occurs only for the monomeric LC whereas the covalently linked P-LC dimer is protected from proteolysis. Thus, a direct link between quaternary structure and proteolytic susceptibility seems to exist. Free light chains (FLCs) can exist both as monomers and as homodimers - so-called Bence-Jones dimers - in vivo, although the term Bence-Jones protein refers rather to urinary LCs^43,44^. Regarding homodimerization and oligomerization, there are differences between lambda and kappa LCs, for instance concerning homodimer affinity although this is still not well understood^45–48^. Our in vitro data suggest that for FOR005, proteolytic cleavage has to occur to allow the amyloid formation of the V_L_ and that disulfide-bridged P-LC homodimers are not susceptible to proteolysis. In consequence, we postulate that impaired dimer association in the endoplasmic reticulum of the plasma cell is a critical step in the disease pathway since it likely leads to enhanced secretion of P-LC monomers, which appear to be the decisive species. Accordingly, we found that the mutations—especially V136G—affect the homodimer association of the FOR005 LC to a large extent. While the single, isolated V_L_ and C_L_ domains are not able to dimerize at the concentrations investigated, the full-length LCs exhibited concentration-dependent monomer–dimer equilibria. This demonstrates that LC dimerization depends on both the V_L_–V_L_ interface and the C_L_–C_L_ interface which are interdependent and can act cooperatively^40^. The dimerization propensities of the P-LC (K_D_ = 62.92 µM) and the G-LC V136G (K_D_ = 53.67 µM) were considerably lower than those of the G-LC (K_D_ = 6.09 µM) and the P-LC G136V (K_D_ = 12.97 µM) which shows the importance of the natively folded C_L_ domain for proper LC dimer association. Previous studies had demonstrated the importance of homodimerization in AL amyloidosis and it is known to protect LCs and V_L_s from amyloid formation^49–51^. Therefore, the V136G mutation in the P-LC pushes the protein toward the amyloid pathway by strongly diminishing the dimerization propensity and, thereby, abrogating an important protective feature of the LC.

**Fig. 7:**
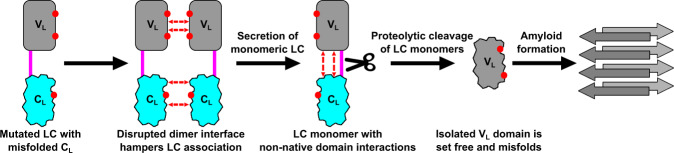
Schematic representation of the mechanistic steps in the disease pathway of FOR005. The V_L_ domain is shown in gray, the C_L_ in cyan, and the linker connecting the two domains is drawn in magenta. The point mutations are indicated as red dots on the LC domains, the dashed red arrows represent nonnative interactions or disrupted native interactions, respectively.

The preferential cleavage of LC monomers by proteases as compared to LC homodimers has already been implied in a previous study by Morgan et al.^52^. Furthermore, secretion of monomeric LCs from plasma cells even in healthy individuals has also been reported^32^. To integrate our findings for FOR005 into a general, stepwise disease mechanism, we propose the following scenario: (1) mutation in the C_L_ domain together with V_L_ mutations disrupt the LC dimer interface, most likely leading to the secretion of LC monomers from plasma cells; (2) these LC monomers are prone to endoproteolysis since the mutations in V_L_ and C_L_ weaken the stabilizing interdomain interactions and domain cooperativity; (3) through proteolytic processing, the highly dynamic and aggregation-prone V_L_ is set free and is now able to form amyloid fibrils since protection by the C_L_ is abolished. The stepwise disease mechanism is summarized in a pathway model in Fig. 7.

However, these proposed mechanistic steps also give rise to new, open questions regarding the disease pathway. Secretion of LC monomers from plasma cells is a known phenomenon, yet our data imply a partial unfolding of the C_L_. It is unknown how and why such a partially unfolded LC can escape ER quality control (ERQC) and subsequent degradation^8^. In this context, pharmacological targeting of the ER protein homeostasis network has previously been shown to compensate for the failed cellular retention of partially folded LCs in AL amyloidosis^53^. Furthermore, the question arises which protease(s) is/are involved in the cleavage of the monomeric LCs in the bloodstream and why the destabilized V_L_ is not degraded or subjected to renal clearance afterward. Accordingly, interactions with plasma proteins and components of extracellular matrices likely play a key role in the onset of AL amyloidosis^54,55^. Additionally, our findings shed light on another problem: in the case of FOR005, the decisive species seems to be the LC monomer since it is highly susceptible to proteolytic cleavage due to the loss of protective stabilization by LC homodimerization. Thus, the recently employed therapeutic approach to stabilize dimeric LCs by small molecules—while elegant and effective for dimers—could potentially be rendered ineffective in a case like FOR005 where monomeric LCs likely need to be targeted instead^32,56–58^. In the future, these and other questions in the context of the onset and progression of AL amyloidosis need to be addressed to gain the further mechanistic insight required for progress in AL diagnosis and therapeutics.

## Methods

All chemicals were purchased from Sigma-Aldrich or VWR unless stated otherwise.

### Sequence and structure analysis

The sequence of FOR005 P-LC obtained from cDNA sequencing was previously reported by Annamalai et al. (GenBank: KX290463)^9^. The corresponding germline light chain sequence, FOR005 G-LC, was determined using NCBI IgBLAST (https://www.ncbi.nlm.nih.gov/igblast/), the international immunogenetics information system (http://www.imgt.org/), and the abYsis database (http://www.abysis.org/abysis/)^16–18^. In the Kabat numbering system for antibodies, the investigated mutation (V136G) is located at position 133. The difference between the Kabat system and the chronological residue number results from an N-terminal glycine residue in our constructs (due to cloning with NcoI) and the fact that N97 and H98 are listed as insertions (i.e., N96A and H96B) at the V-J-junction in the Kabat system^19^. A homology model for the three-dimensional structure of P-LC was obtained from SWISS-MODEL, and the crystal structure of 5BV7 (96.3% sequence identity) was chosen as the modeling template^21^.

### Cloning, mutagenesis, protein expression, and purification

Synthetic DNA constructs in expression vector pET28b(+) were obtained from Invitrogen (Carlsbad, USA). Mutant variants were cloned by site-directed mutagenesis using NEBaseChanger. In all proteins, the C-terminal cysteine residue was replaced by serine. This cysteine usually builds up an intermolecular disulfide bridge in covalent LC homodimers^22^. However, we also produced one construct with Cys214 reconstituted to obtain covalently linked P-LC homodimers (Bence-Jones protein). Primers were purchased from Eurofins Genomics (Ebersberg, Germany). Polymerase chain reactions using Q5 polymerase (New England Biolabs, Frankfurt, Germany) and subsequent KLD enzyme reactions were performed according to the manufacturer’s protocol. Plasmid sequencing was performed by Eurofins Genomics (Ebersberg, Germany). The recombinant proteins were expressed as insoluble inclusion bodies using *E. coli* BL21 (DE3)-star cells at 37 °C overnight after induction with 1 mM IPTG. After harvesting the bacteria, inclusion bodies were prepared as previously described^33^. The LCs were refolded and purified similarly to the method we have previously described^10^. Briefly, the inclusion bodies were solubilized in PBS containing 8 M urea and 0.1% β-mercapto ethanol at room temperature for 4–6 h, and then stepwise dialysis was performed at 10 °C to refold the LCs by sequentially decreasing the urea concentration. The applied dialysis steps were 5 M urea, pH 8.0, then 3 M urea, pH 8.5, and 1 M urea in PBS, pH 7.4, followed by a final dialysis step using only PBS pH 7.4. Between the steps with 5 and 3 M urea, anion exchange chromatography using Q-Sepharose (GE Healthcare, Uppsala, Sweden) was performed with a running buffer containing 5 M urea. The protein concentration for refolding was adjusted to 0.5 mg/ml. After the final dialysis against PBS, size-exclusion chromatography using a Superdex75 column (GE Healthcare, Uppsala, Sweden) was performed as a polishing step.

In the case of the C_L_ domains and the P-LC dimer (Bence-Jones protein), a different refolding protocol, also similar to a method previously described by our lab, was applied^33^. Briefly, the inclusion bodies were solubilized using 8 M urea and 0.1 β-mercapto ethanol, followed by dialysis against 5 M urea-containing buffer and subsequent anion exchange chromatography. Then, refolding was performed by dialyzing against 50 mM Tris (pH 8.0), 100 mM L-Arginine, 1 mM oxidized glutathione, and 0.5 mM reduced glutathione at 10 °C overnight. For the P-LC dimer, 2 mM of oxidized glutathione was used for refolding to enhance the formation of disulfide-linked P-LC homodimers. Afterward, a final dialysis step against PBS and size-exclusion chromatography was performed. The identity and purity of the proteins were checked by SDS-PAGE and mass spectrometry.

### Circular dichroism spectroscopy

Circular dichroism was measured on a JASCO J-1500 CD spectrometer (JASCO, Großumstadt, Germany). Far-UV spectra were recorded in a 1-mm quartz cuvette at 20 °C from 260 to 200 nm using 0.1 mg/ml protein diluted in PBS. Near UV spectra were recorded in a 2-mm quartz cuvette at 20 °C from 260 to 320 nm using 0.5 mg/ml protein in PBS. Thermal transitions were recorded from 20 to 80 °C at 205 nm using a heating rate of 1 °C/min. The melting temperature represents the transition midpoint (50% unfolded) which was determined using the Boltzmann fit. After thermal denaturation, the samples were cooled down to 20 °C and equilibrated for at least 1 h before FUV-CD spectra of the refolded proteins were recorded at 20 °C to investigate the reversibility of unfolding. All data were obtained from two independent measurements (*n* = 2) and then averaged, except for the FUV spectra of refolded proteins after thermal denaturation which were only measured once.

### Differential scanning calorimetry

Differential scanning calorimetry was performed as two independent experiments (*n* = 2) for every protein on a MicroCal PEAQ DSC (Malvern Panalytical, Malvern, UK). Thermograms were obtained from 20 to 80 °C at a scan rate of 1 °C/min and with a protein concentration of 1.0 mg/ml in PBS. The raw data were analyzed with the PEAQ DSC software (Malvern Panalytical) by applying a progressive baseline fit (lower cutoff: approximately at 25–30 °C / upper cutoff: 70–80 °C). Subsequently, the baseline-corrected data was fitted with a non-two-state fitting model to derive thermodynamic parameters. The presented thermograms represent the mean of the two individual measurements. Reported values for T_m_ and ΔH also represent the mean of the two experimental values.

### Thioflavin T binding kinetics

Protein stock solutions were thawed and centrifuged in an Optima MAX-E ultracentrifuge (Beckman, Krefeld, Germany) for 3–4 h at 45,000 rpm to remove aggregates. All assay components were filtered through a 0.22-µm filter (Merck, Darmstadt, Germany) before the samples were prepared. Thioflavin T assays were performed in triplicates (*n* = 3) over a time span of 2 weeks using 15 µM protein, 7.5 µM ThT, 0.05% sodium azide, 0.5 mM SDS, pH 7.4 or 6.4, at 37 °C. Samples of 200 µl were incubated in 96-well Nunc plates (Thermo Fisher, Roskilde, Denmark) sealed with adhesive microplate film (VWR, Radnor, USA) under continuous orbital shaking in a Tecan Genios plate reader (Tecan Group Ltd., Männedorf, Switzerland). The shaking intensity was set to high. The excitation wavelength was 440 nm, and the emission wavelength was 480 nm. The reported kinetics represent the mean of three measurements with standard deviation.

### Transmission electron microscopy

Samples of 5 µl from finished ThT assays were loaded onto activated copper grids (200 mesh) for 1 min. The grids were then washed with 20 µl H_2_O and stained with 5 µl 2% uranyl acetate for 1 min. A filter paper was used to remove excess solution from the grids. TEM micrographs were obtained on a JEOL JEM 1400-plus transmission electron microscope (JEOL Germany GmbH, Freising, Germany) at 120 kV. The data/images are derived from single measurements (*n* = 1).

### Acrylamide quenching

To assess the accessibility of the buried tryptophan residues by the quenching agent acrylamide, proteins were incubated at 15 µM with increasing concentrations of acrylamide (0–1 M in 0.1 M steps). Incubation was carried out overnight at room temperature in the dark. Fluorescence was measured in duplicates (*n* = 2) in a Tecan Infinite Nano plate reader at 25 °C using Greiner UV-Star 96-well plates (Greiner Bio One, Kremsmünster, Austria). There was only one exception, namely the sample P-LC G136V at 1 M acrylamide for which only one measurement was obtained. The excitation wavelength was 292 nm and emission spectra were recorded between 315 and 380 nm. The wavelength of maximum emission of the native sample (no acrylamide) was determined and the respective quenching rate was calculated (F_0_/F), where F_0_ is the fluorescence intensity without a quenching agent. The mean values of the two experiments were plotted against the acrylamide concentration. The slope of the linear fit represents the Stern–Volmer constant (k_sv_). Outliers were excluded from the data fit as indicated.

### Red-edge excitation shift (REES) fluorescence spectroscopy

REES fluorescence can be applied to investigate the folding and dynamics of proteins by performing 3D excitation-emission scans^29^. The 3D scans were recorded in two independent experiments (*n* = 2) on a JASCO FP8500 spectrofluorimeter (equipped with a Peltier element) at 25 and 37 °C, respectively. The protein concentration was 20 µM in PBS, denatured samples contained 6 M urea, and all samples were equilibrated at the respective temperature for ~3 h prior to measurement. Excitation wavelengths went from 280 to 300 nm in 1 nm steps and the single emission spectra were recorded between 315 and 400 nm. The reported data represent the mean of two measurements after buffer subtraction. The center of spectral mass (CSM) was determined by the following equation

1 $${{{{{\rm{CSM}}}}}}=\sum ({{{{{{\rm{f}}}}}}}_{{{{{{\rm{i}}}}}}}* {\lambda }_{{{{{\rm{em}}}}}})/\sum ({{{{{{\rm{f}}}}}}}_{{{{{{\rm{i}}}}}}})$$

where f_i_ represents the fluorescence intensity and *λ*_em_ is the emission wavelength.

### Molecular dynamics (MD) simulations

All molecular dynamics (MD) simulations were carried out once (*n* = 1) and analyzed using the Amber18 molecular simulation package^59^. Simulations were performed starting from the P-CL (G136) domain (residues 110–212 of the P-LC structure) and the G-CL (V136) structures. Each protein was solvated in TIP3P water^60^ in a periodic octahedral box with a minimum distance of protein atoms to the box boundary of 10 Å. The ff14SB force field^61^ was employed and Na^+^ and Cl^−^ ions were added to neutralize the system and reach an ion concentration of 0.15 M. Energy minimization of each system was performed with the sander module of Amber18 (2500 minimization cycles). The systems were heated in steps of 100 K (50 ps per step) to a final temperature of 310 K with the solute non-hydrogen atoms harmonically restrained to the start structure. All bonds involving hydrogen atoms were kept at an optimal length. In the additional four steps, the harmonic restraints were removed stepwise. For the subsequent production simulations, hydrogen mass repartitioning (HMR) was employed allowing a time step of 4 fs (instead of 2 fs used during heating and equilibration). Unrestrained production simulations were extended to 1.6 µs for each system. Coordinates were saved every 8 ps. Root-mean-square deviation (RMSD) and root-mean-square fluctuations (RMSF) were performed using the cpptraj module of Amber18. Umbrella sampling (US) simulations were performed using the center of the mass distance between backbone atoms of residues 135–137 (including either the V136 or G136 residue) and residues 120–121 (belong to the N-terminal β-strand of the C_L_ domain). The reference distance in the quadratic restraining potential (force constant 2.5 kcal mol^−^^1^Å^−^^2^) varied from 4.5 to 9.0 Å in steps of 0.5 Å. Sampling in each US interval was performed using 5 ns equilibration followed by 30 ns data gathering. The associated potential of mean force (PMF), i.e., the free energy change along the reaction coordinate, was calculated using the weighted-histogram analysis method (WHAM).

### Limited proteolysis

The proteins were diluted to 0.3 mg/ml in 100 mM Tris, 100 mM NaCl, 10 mM CaCl_2_, pH 7.8, and incubated at room temperature (around 22 °C) with trypsin using a final substrate/enzyme ratio of 20/1 (w/w) and with proteinase K at a final substrate/enzyme ratio of 250/1 (w/w). Samples were taken from the reaction at the indicated time points and mixed with PMSF (final concentration 2 mM) and 5x Lämmli buffer to stop the proteolytic degradation. SDS-PAGE samples were run on a SERVA Prime 4 - 20% gel. All limited proteolysis experiments were performed as duplicates (*n* = 2). Densitometric analysis of the SDS gels was performed using NIH ImageJ and the data points represent the mean of two measurements.

### LC-MS

To identify fragments created by limited proteolysis, ESI-TOF mass spectrometry was applied. Limited proteolysis experiments with monomeric P-LC and G-LC V136G were carried out as single experiments (*n* = 1) as described above with trypsin and proteinase K. After ~2 min of incubation, a sample from the proteolysis reaction was taken and mixed with an excess of PMSF to stop the degradation. Subsequently, the samples were measured on a Synapt XS ESI-TOF mass spectrometer (Waters Corp.) equipped with an ACQUITY HPLC system (Waters Corp.). Samples were run on a C4 column and a 0–95% acetonitrile gradient was applied. The raw data were analyzed with MassLynx software (Waters Corp.) and the resulting fragments in the deconvoluted mass spectra were analyzed with ExPASy ProtParam and FindPept.

### Analytical ultracentrifugation

AUC measurements at different protein concentrations (in PBS) were performed in an Optima-AUCI centrifuge (Beckman, Krefeld, Germany) equipped with absorbance optics. The respective sample concentrations were adjusted according to the absorbance value at 280 nm (i.e., A_280_ = 0.25/0.5/1.0/1.5/2.0/2.5), and the corresponding molar concentrations were calculated. Due to the detector limit of the absorbance optics in our XL-I ultracentrifuge, A_280_ = 2.5 was the maximum protein concentration that could be analyzed. A volume of 350 µl sample solution was loaded into the assembled cells which are equipped with quartz windows and 12-mm-path-length charcoal-filled epon double-sector centerpieces. All measurements were carried out at 42,000 rpm and 20 °C with an eight-hole Beckman-Coulter AN50-ti rotor. Sedimentation was continuously scanned with a frequency of 100 s, a data resolution of 10 µm, and was monitored at 280 nm. SEDFIT in continuous c(S) distribution mode was used for data analysis. The peak areas of the AUC runs were integrated and the dimer fraction was plotted against the respective total protein concentration (micromolar). The apparent K_D_ of noncovalent LC dimerization was estimated by applying a Hill function fit which included a dimer fraction of 0 at 0 µM protein concentration. All data are derived from single measurements (*n* = 1) at each LC concentration.

### Reporting summary

Further information on research design is available in the Nature Portfolio Reporting Summary linked to this article.

## Supplementary information

Supplementary information

Description of Additional Supplementary Files

Supplementary data 1

Reporting summary

## Data Availability

All data are contained within the main article and Supplementary Information. The source data behind all figures and Supplementary figures are included in Supplementary Data 1. Further information can be obtained from the authors upon reasonable request.
