# Supplementary material for: A constant domain mutation in a patient-derived antibody light chain reveals principles of AL amyloidosis
Source: Commun Biol. 2023 Feb 23;6:209. doi: 10.1038/s42003-023-04574-y (PMC9950467; doi:10.1038/s42003-023-04574-y)
Supplement: Supplementary file 1 — Supplementary information [file 42003_2023_4574_MOESM1_ESM.pdf]

Supplementary Information

A constant domain mutation in a patient-derived antibody light chain reveals principles of AL amyloidosis

Georg J. Rottenaicher[1,2], Ramona M. Absmeier[1,2], Laura Meier[1,2], Martin Zacharias[1,2], Johannes Buchner[1,2*]

[1]Center for functional Protein Assemblies, Technical University Munich, Ernst-Otto-Fischer-Str. 8, 85748 Garching, Germany

[2]Department of Biosciences, TUM School of Natural Sciences, Technical University Munich, Boltzmannstr. 10, 85748 Garching, Germany

*Corresponding author: Johannes Buchner, johannes.buchner@tum.de

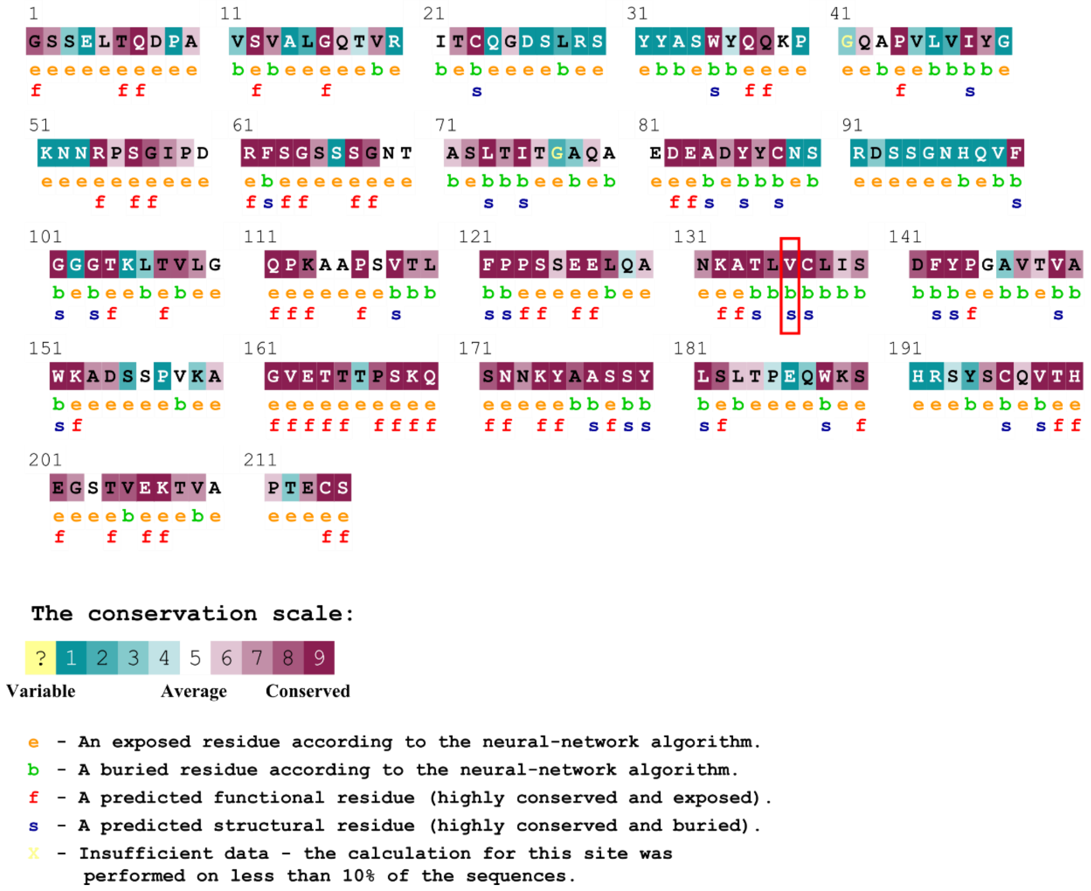

Supplementary Figure 1: Analysis of the G-LC sequence using the Consurf web tool indicates integral role of V136. The red box marks the valine residue at position 136 that is mutated to glycine in P-LC. As indicated by the conservation scale, residues colored in cyan have a low conservation degree and residues in white have an average conservation degree. Purple residues are highly conserved. According to the Consurf algorithm, valine 136 is highly conserved and buried inside the protein core of G-LC.

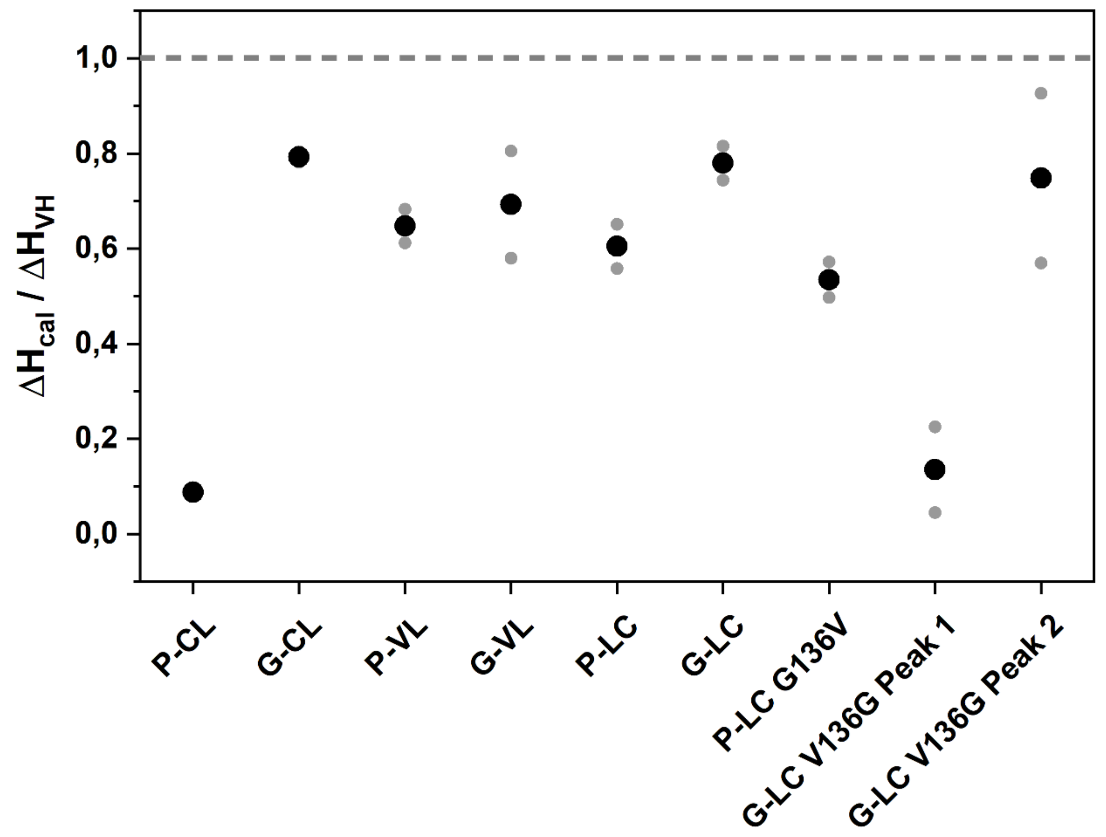

Supplementary Figure 2: ΔH_{cal} / ΔH_{VH} ratios imply the population of partially unfolded states. As indicated, the only protein that exhibited two distinct transitions was G-LC V136G. All other proteins either unfolded in a single, cooperative transition or the two transition temperatures of the individual domains were too close to each other to be resolved by DSC. Theoretically, the fraction of ΔH_{cal} / ΔH_{VH} is 1 for an ideal two-state unfolding event (indicated by the dashed grey line). The stronger a value deviates from 1, the more likely partially folded states are being populated among the folding trajectories. Individual data points are shown as gray dots, the mean of the two independent measurements (n = 2) is shown as black dots.

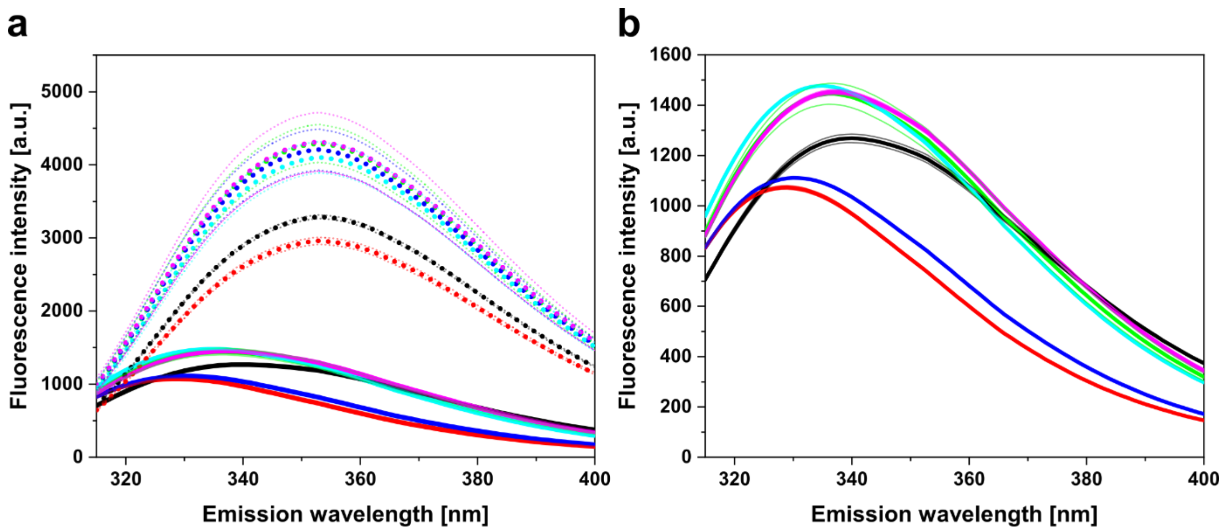

Supplementary Figure 3: Fluorescence spectra in the unfolded and native state show that P-CL is partially unfolded. a Fluorescence spectra of proteins denatured with 6 M urea (dotted lines) and native proteins (solid lines) in PBS pH 7.4. **b** Spectra of the native proteins. Fluorescence spectra were recorded between 315-400 nm with an excitation wavelength of 295 nm at 25 °C on a Jasco FP-8500 spectrofluorometer. Coloring: P-CL in black, G-CL in red, P-LC in green, G-LC in blue, P-LC G136V in cyan, G-LC V136G in magenta. The mean of duplicates (n = 2) is shown as thick lines, the individual spectra are shown as narrow lines.

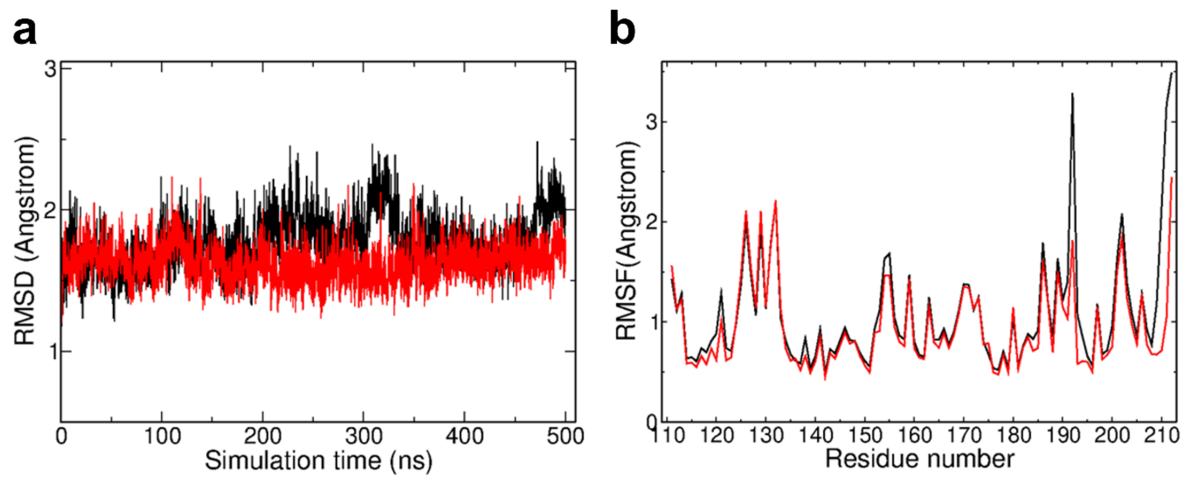

Supplementary Figure 4: Molecular dynamics simulation of P-CL and G-CL. a Root mean square deviation (RMSD of non-hydrogen atoms) from the start structure during 500 ns MD simulation time (n = 1; Coloring: P-CL in black, G-CL in red). **b** Root mean square fluctuations of P-CL and G-CL residues with respect to mean structure recorded during MD-simulations (same coloring as in a).

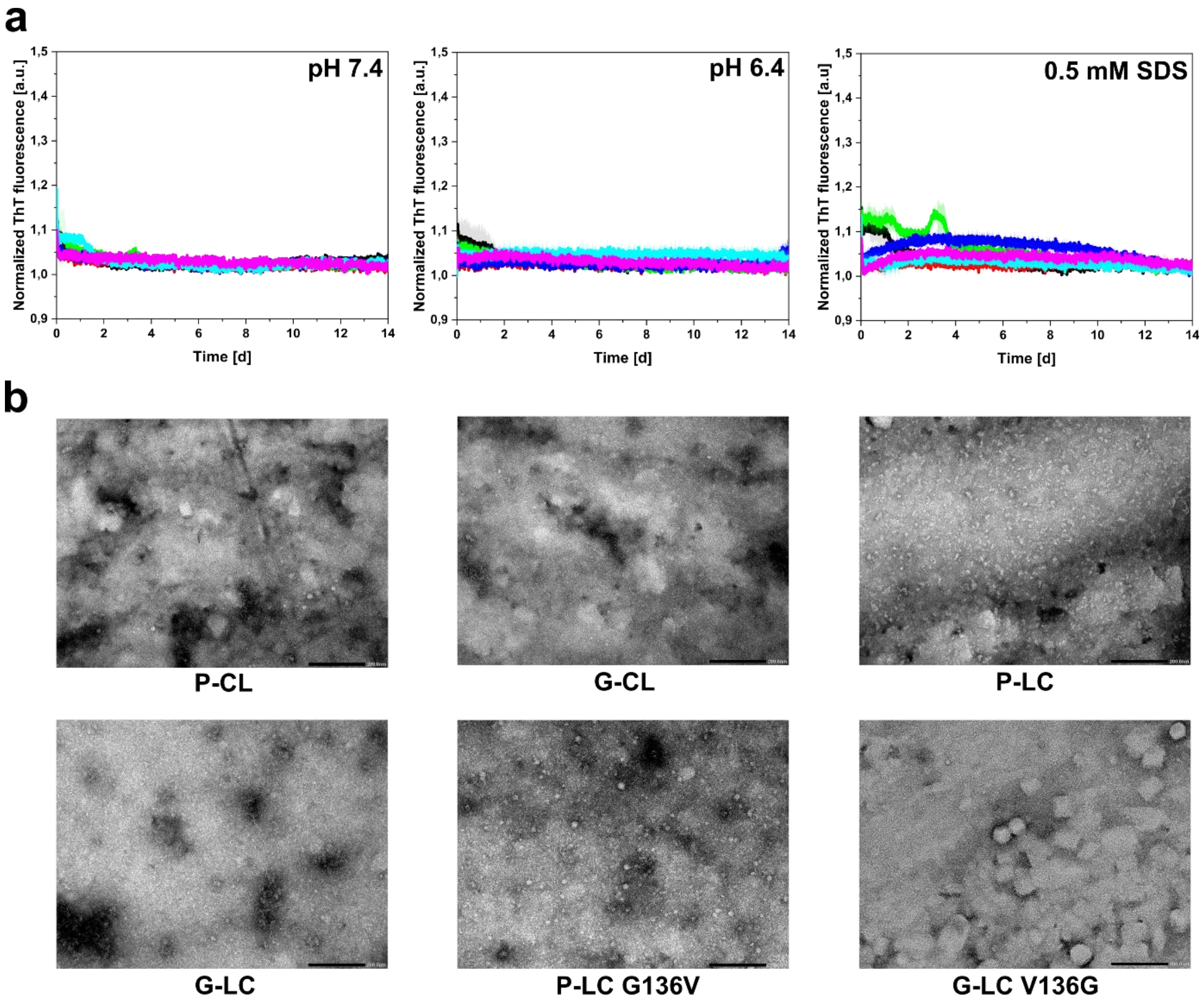

Supplementary Figure 5: ThT binding kinetics and TEM micrographs demonstrate that the C_Ls and all LCs are resistant to amyloid formation. a ThT kinetics were obtained as triplicates (n = 3) over two weeks under continuous shaking in a microplate reader at 37 °C and either pH 7.4 (left panel), pH 6.4 (middle panel), or in the presence of 0.5 mM SDS (right panel). The data represents mean of three replicates with standard deviation shown as respectively colored shadows. **b** TEM micrographs (n = 1) of finished ThT assays at pH 7.4 show the presence of amorphous aggregates and confirm the absence of amyloid fibrils. The black scale bars in the lower right corner of every micrograph represent 200 nm. Coloring: P-CL in black, G-CL in red, P-LC in green, G-LC in blue, P-LC G136V in cyan, G-LC V136G in magenta.

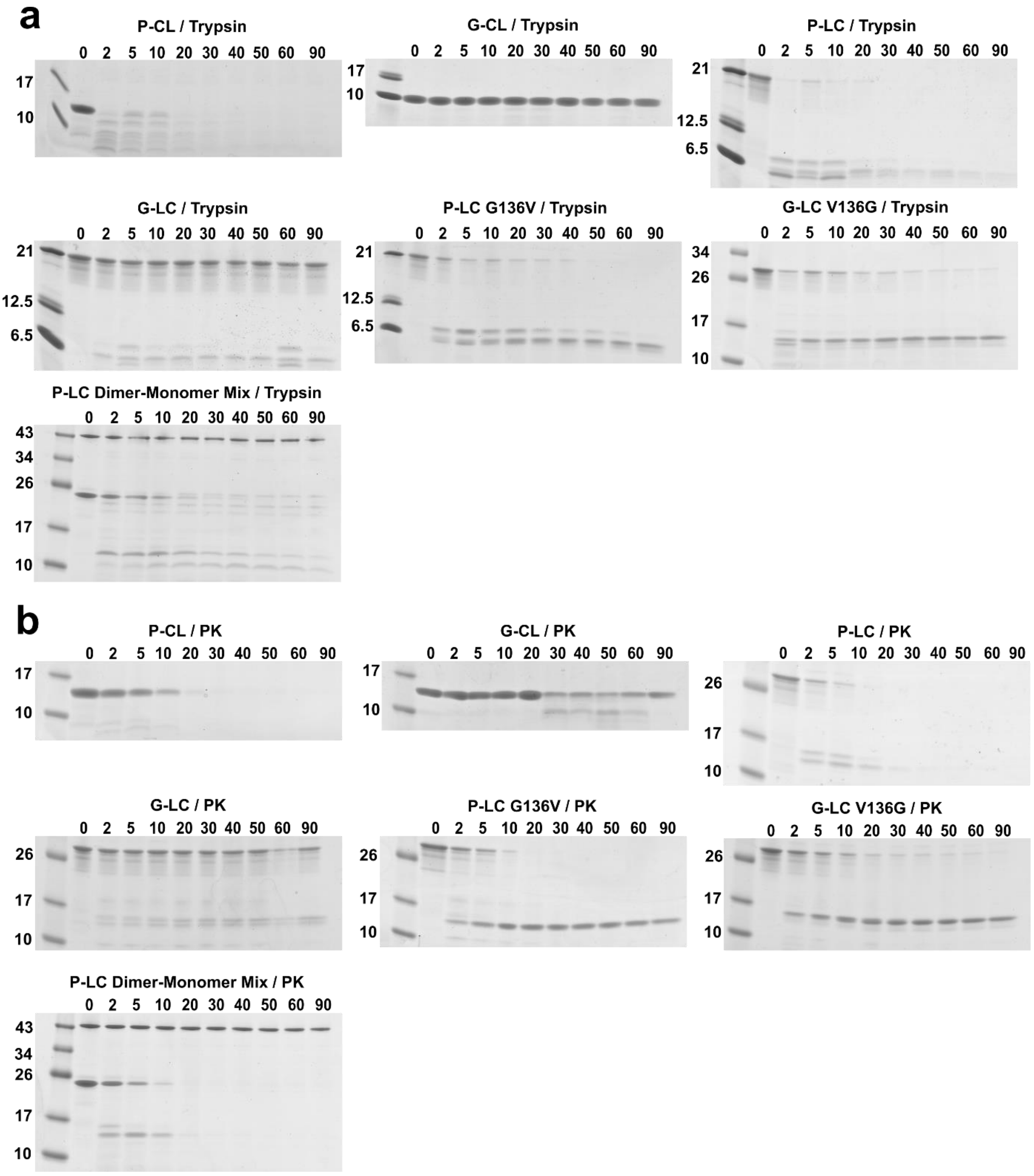

Supplementary Figure 6: SDS-PAGE gels of limited proteolysis experiments with trypsin and proteinase K show the differences in proteolytic susceptibility. a For trypsin, a 20-fold substrate excess was used. **b** For experiments with proteinase K a 250-fold substrate excess was used. Samples were run on SERVA TG Prime 4-20 % SDS gels with 25 mA. Limited proteolysis experiments were carried out in duplicates (n = 2), however, only one gel is shown per investigated protein. Experiments were performed at room temperature.

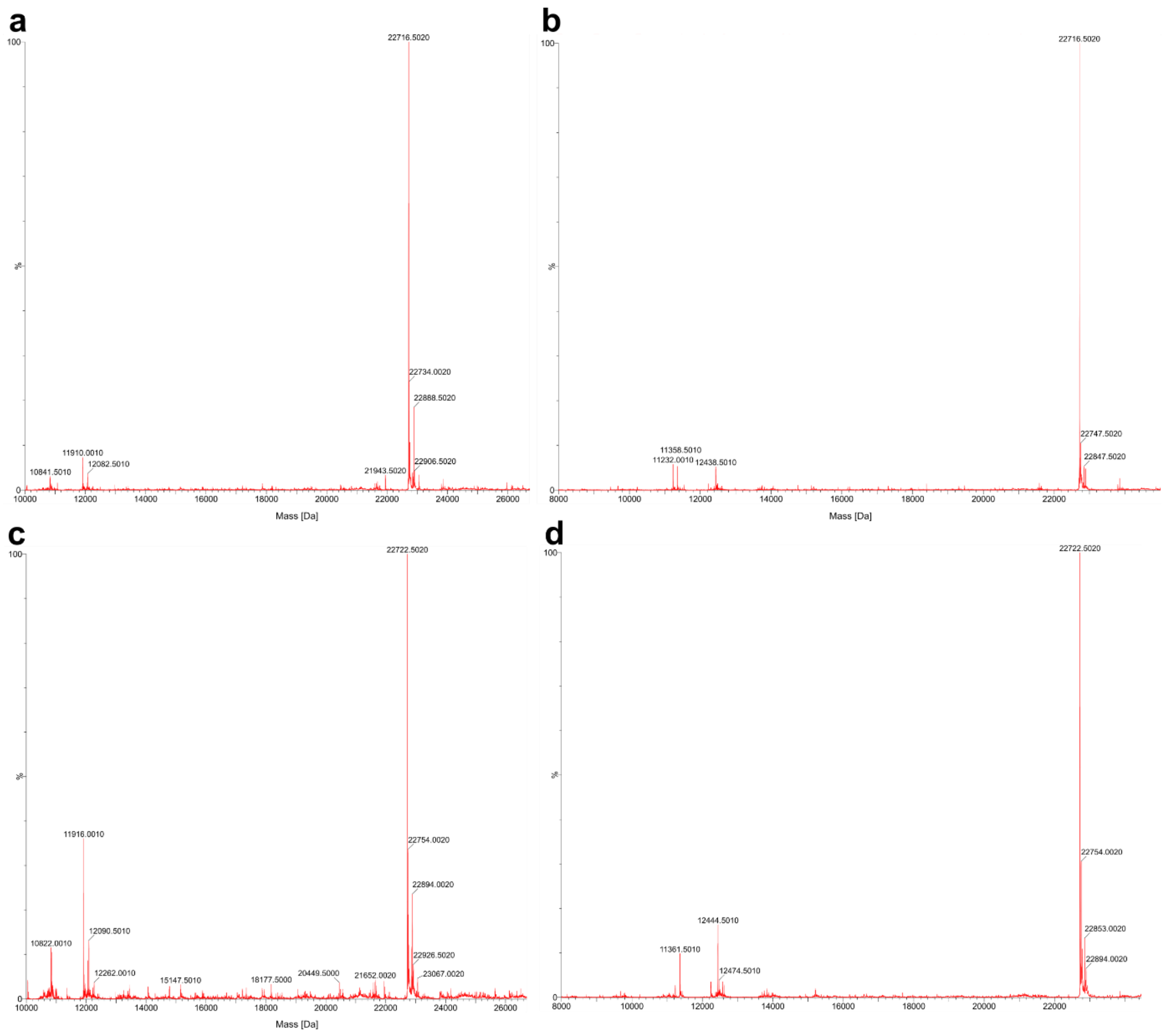

Supplementary Figure 7: Identification of fragments generated by limited proteolysis using full-length ESI-TOF mass spectrometry. a P-LC monomer cleaved with trypsin. **b** P-LC monomer cleaved with proteinase K. **c** G-LC V136G cleaved with trypsin. **d** G-LC V136G cleaved with proteinase K. For trypsin, a 20-fold substrate excess was used, whereas for proteinase K a 250-fold substrate excess was used. The proteolysis experiments were carried out for approximately two minutes at room temperature before the reaction was stopped by adding an excess of PMSF. Peaks around 22.7 kDa represent the residual full-length LC. The peaks between 10 – 13 kDa are the cleaved fragments which were analyzed by ExpPASy FindPept and ProtParam. The fragment analysis is shown in Supplementary Table 1. All data represent single measurements (n = 1).

Supplementary Table 1: ESI-TOF MS analysis of fragments from limited proteolysis. The limited proteolysis samples were subjected to full-length ESI-TOF mass spectrometry. The resulting fragments in the deconvoluted mass spectra were analyzed with ProtParam and FindPept. The fragments which result from the suspected cleavage in the linker between V_L and C_L are highlighted in green. These fragments most likely correspond to the respective mass values, although we cannot identify them completely unambiguously. All data represents single measurements (n = 1).

Mass [Da]	Fragment	Comments
	**P-LC + Trypsin**	
**10841.5**	1-103	Unspecific cleavage of V_L shortly before linker
	4-105	Specific cleavage of V_L in linker region; first 3 AAs missing
	34-136	Unspecific cleavage in CDR1 and C_L
	77-180	Unspecific cleavage in FR3 and C_L
	85-187	Unspecific cleavage in FR3 and C_L
	96-197	Unspecific cleavage in CDR3 and C_L
	109-210	Residual C_L domain; unspecific cleavage of linker
**11910.0**	1-113	Specific cleavage of V_L at K113 in linker region
**12082.5**	1-113 + PMSF	Possible PMSF adduct (MW: 174.2 Da) of cleaved V_L
	29-142	Unspecific cleavage in CDR1 and C_L
	39-154	Unspecific cleavage in FR2 and C_L
	89-202	Unspecific cleavage in CDR3 and C_L
	95-208	Unspecific cleavage in CDR3 and C_L
	**P-LC + Proteinase K**	
**11232.0**	9-115	V_L cleaved in linker region (specific); first 8 AAs missing
	10-116	V_L cleaved in linker region (unspecific); first 9 AAs missing
	19-124	Unspecific cleavage in FR1 and C_L
	29-134	Unspecific cleavage in CDR1 and C_L
	42-149	Unspecific cleavage in FR2 and C_L
	66-174	Unspecific cleavage in FR3 and C_L
	91-196	Unspecific cleavage in CDR3 and C_L
	110-215	Residual C_L domain after specific cleavage in linker
**11358.5**	41-150	Unspecific cleavage in FR2 and C_L
	43-151	Unspecific cleavage in FR2 and C_L
	72-180	Specific cleavages in FR3 and C_L
	74-182	Unspecific cleavage in FR3 and C_L
	76-184	Unspecific cleavage in FR3 and C_L
	79-186	Unspecific cleavage in FR3 and C_L
	80-187	Unspecific cleavage in FR3 and C_L
	91-197	Unspecific cleavage in CDR3 and C_L
**12438.5**	1-119	V_L after unspecific cleavage in linker
	22-139	Specific cleavages in FR1 and C_L

Continued Supplementary Table 1

**G-LC V136G + Trypsin**		
**10822.0**	29-130	Unspecific cleavage in CDR1 and C_L
	31-132	Unspecific cleavage in CDR1 and C_L
	36-139	Unspecific cleavage in FR2 and C_L
	49-152	Unspecific cleavage in FR2 and C_L
	53-157	Unspecific cleavage in CDR2 and C_L
	57-162	Unspecific cleavage in FR3 and C_L
	89-191	Unspecific cleavage in CDR3 and C_L
	110-211	C_L after unspecific linker cleavage
**11916.0**	1-113	V_L after specific cleavage in linker (K113)
	52-167	Unspecific cleavage in CDR2 and C_L
	61-175	Unspecific cleavage in FR3 and C_L
**12090.5**	1-113 + PMSF	Possible PMSF adduct (MW: 174.2 Da) of cleaved V_L
	29-142	Unspecific cleavage in CDR1 and C_L
	63-180	Unspecific cleavage in FR3 and C_L
	67-183	Unspecific cleavage in FR3 and C_L
	85-197	Unspecific cleavage in FR3 and C_L
**G-LC V136G + Proteinase K**		
**11361.5**	3-109	Specific cleavage of V_L in linker region; first 2 AAs missing
	15-121	Specific cleavages at A14 and shortly after linker at start of C_L
	35-142	Unspecific cleavage in CDR1 and C_L
	51-160	Unspecific cleavage in CDR2 and C_L
	64-174	Unspecific cleavage in FR3 and C_L
**12444.5**	1-119	V_L after unspecific cleavage in linker region
	21-138	Unspecific cleavage in FR1 and C_L
	37-156	Unspecific cleavage in FR2 and C_L
	50-170	Unspecific cleavage in FR2 and C_L
	59-179	Unspecific cleavage in FR3 and C_L
**12474.5**	1-119 + 30 Da	Possible formylation or oxidation of cleaved V_L

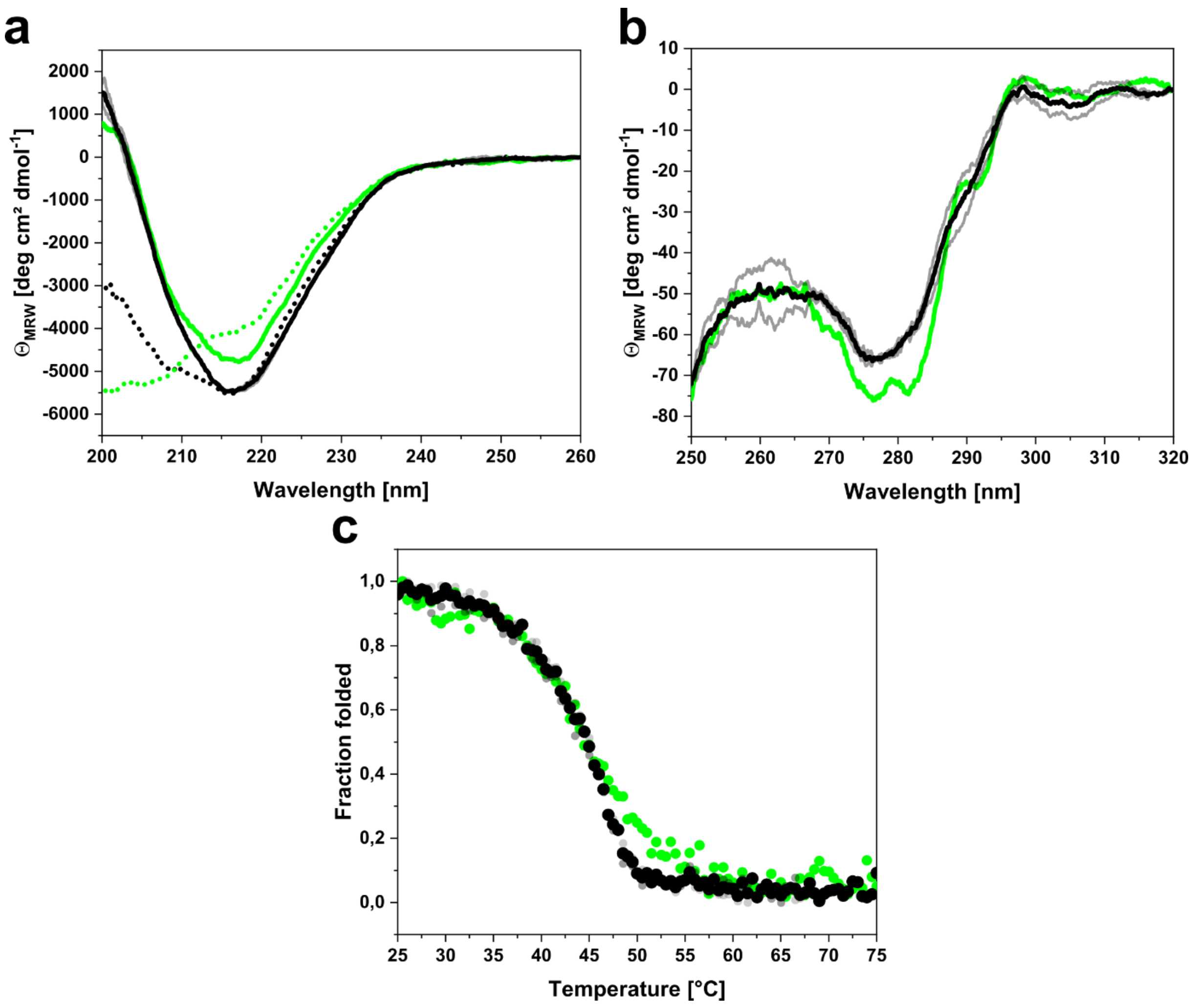

Supplementary Figure 8: CD spectroscopy of the P-LC dimer/monomer mixture reveals dimerization-induced differences in folding and stability. **a** FUV-CD spectrum of the native (solid black line) and refolded (dotted black line) P-LC dimer/monomer mixture. Respective spectra of the regular P-LC monomer are shown in green (mean of two curves). The individual replicates of P-LC dimer/monomer mix are shown in grey. The different spectra indicate altered secondary structure and partially reversible unfolding of P-LC due to covalent dimerization. **b** NUV-CD spectrum of the P-LC monomer/dimer mix (black) and the P-LC monomer (green; mean of two curves). The individual replicates of P-LC dimer/monomer mix are shown in grey. **c** Thermal denaturation of P-LC monomer/dimer mix (black dots) followed by CD spectroscopy at 205 nm. The individual data points of P-LC dimer/monomer mix are shown in grey. The melting temperature T_m of the dimer/monomer mix was determined to be 44.24 °C by Boltzmann fit. Therefore, the dimer/monomer mix exhibits a slightly higher apparent thermal stability than the completely monomeric P-LC (mean shown as green dots; T_m = 41.38 °C). All CD data is derived from two independent measurements (n = 2).

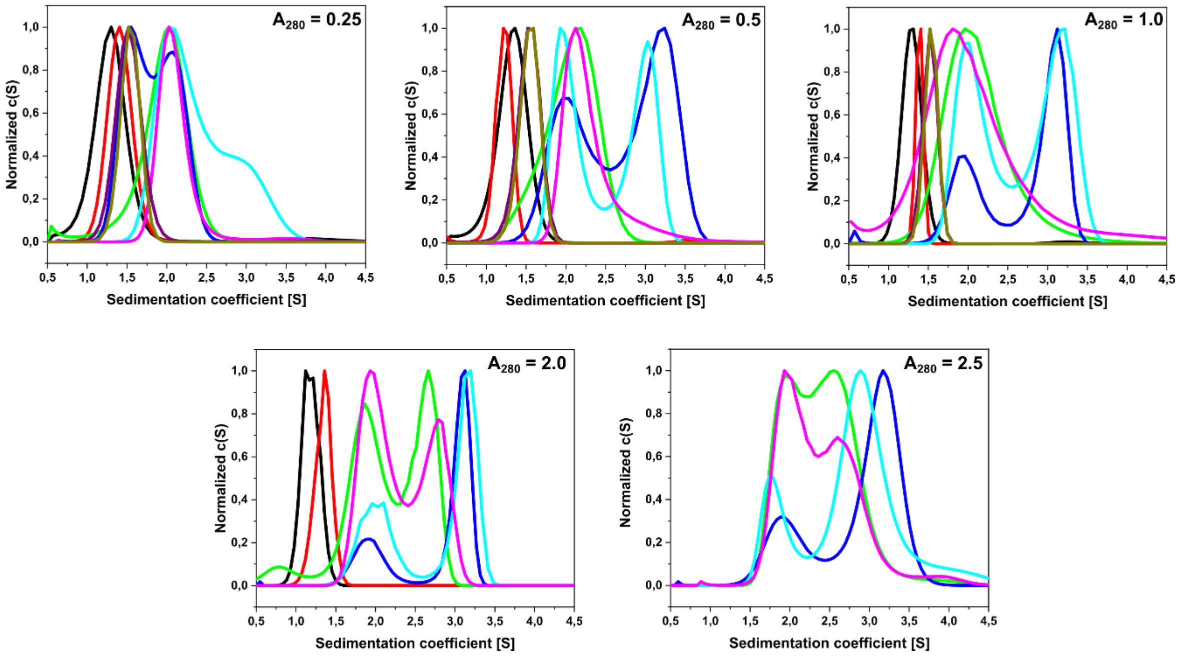

Supplementary Figure 9: Analytical ultracentrifugation shows the differences in dimerization propensity between the investigated variants. AUC runs at A_{280} = 0.25/ 0.5/ 1.0/ 2.0/ 2.5. The run with A_{280} = 1.5 is shown in Figure 5 of the main article. In the last two runs (A_{280} = 2.0/2.5) the single V_L domains were not investigated and in the last run (A_{280} = 2.5) the C_L domains were also neglected. (Coloring: P-CL in black, G-CL in red, P-LC in green, G-LC in blue, P-LC G136V in cyan, G-LC V136G in magenta, P-VL in purple, G-VL in olive). AUC runs were performed at 20 °C and 42 000 rpm. Data represents single measurements (n = 1). For data analysis SEDFIT was used.

Supplementary Table 2: Dimer and monomer fractions derived from AUC measurements at the respective total LC protomer concentrations. Since the single V_L and C_L domains did not show dimerization at the concentrations investigated, they are not included in the table. Data is derived from single measurements (n = 1).

Protein	Absorbance A_{280}	Total LC concentration [µM]	Fraction Dimer	Fraction Monomer
**P-LC**	0	0	0	0
	0.25	8.89	0	1
	0.5	17.61	0	1
	1.0	35.22	0	1
	1.5	52.17	0.085	0.915
	2.0	69.77	0.458	0.542
	2.5	87.21	0.599	0.401
**G-LC**	0	0	0	0
	0.25	7.85	0.475	0.525
	0.5	15.83	0.581	0.419
	1.0	30.81	0.650	0.350
	1.5	47.32	0.685	0.315
	2.0	63.20	0.722	0.278
	2.5	79.00	0.744	0.256
**P-LC G136V**	0	0	0	0
	0.25	8.76	0.142	0.858
	0.5	17.45	0.495	0.505
	1.0	35.15	0.541	0.459
	1.5	52.36	0.590	0.410
	2.0	69.75	0.614	0.386
	2.5	87.24	0.765	0.235
**G-LC V136G**	0	0	0	0
	0.25	7.62	0	1
	0.5	15.81	0	1
	1.0	31.63	0	1
	1.5	47.15	0.061	0.939
	2.0	63.20	0.410	0.590
	2.5	78.98	0.448	0.552
