# Supplementary material for: A constant domain mutation in a patient-derived antibody light chain reveals principles of AL amyloidosis
Source: Commun Biol. 2023 Feb 23;6:209. doi: 10.1038/s42003-023-04574-y (PMC9950467; doi:10.1038/s42003-023-04574-y)
Supplement: Supplementary file 2 — Description of Additional Supplementary Files [file 42003_2023_4574_MOESM2_ESM.pdf]

Description of Additional Supplementary Files

File Name: Supplementary Data

Description: Source data behind all figures and supplementary figures.
